# Cameras in the Hands of Indigenous Youth: Participation, Films, and Nutrition in India

**DOI:** 10.1093/cdn/nzac114

**Published:** 2022-08-19

**Authors:** Nitya Rao, Nivedita Narain, Ghezal Sabir

**Affiliations:** School of International Development, University of East Anglia, Norwich, United Kingdom; Professional Assistance for Development Action, New Dehli, India; Nutrition and food literacy consultant, Aargau, Switzerland

**Keywords:** participatory filmmaking, traditional food knowledge, Aboriginal youth, Santhal, food literacy

## Abstract

**Background:**

Indigenous communities in India have diets that do not fulfill all of their minimum nutritional requirements. Given the unaffordability of healthy diets, these communities rely on common-pool resources to make up for shortfalls in food. Yet, such foods are devalued as “backward,” and accessing them is regulated by unequal gendered roles.

**Objectives:**

To explore the central role of community participation in documenting and transmitting indigenous knowledge about the role of locally available foods in improving dietary diversity.

**Methods:**

Through a participatory action research approach, 10 Santhal youth were trained to make films about a range of locally available foods and other issues of concern to them (Santhal/Santal is a native ethnic group in India). These films were broadcast on a YouTube channel and screened locally. A thematic content analysis of 49 films was undertaken, alongside interviews with the filmmakers and focus group discussions with viewers who attended 4 film screenings.

**Results:**

A majority of the films produced drew on intergenerational and indigenous knowledge about edible plants, insects, and rodents; skills in foraging and preparing food; awareness of the benefits of the food; and sustainability issues across the traditional food systems. The filmmakers initially focused on responding to community needs and showcasing Santhal cultural practices. Their later films began to reflect on aspects of their culture that needed to be preserved, revived, or modified. Audiences noted the relatability and relevance of the provided information, generated ideas and priority themes for further documentation, and expressed the need for revival and modification of certain cultural food practices.

**Conclusion:**

A participatory filmmaking process in the context of community nutrition can enable participants to question unequal power relations by enabling the most marginalized to voice their own perspectives with the support of cameras and filmmaking skills.

## Introduction

The world is not on target to meet Sustainable Development Goal 2 (SDG2), related to food security and nutrition (1). The SDGs are a series of 17 goals adopted by the United Nations in 2015 as a universal call to action to end poverty, protect the planet, and ensure that by 2030 all people enjoy peace and prosperity (2). Several pathways have been identified, including boosting resilience to climatic and economic adversities (3), addressing structural inequalities (4), and driving consumer behavior toward sustainable practices (5). However, an important component of food security and nutrition interventions—the role of community participation—has not been adequately highlighted. This article demonstrates the central role of community participation in promoting sustainable food system practices and *dietary diversity*:

Food insecurity, extracted from the most widely used definition of food security (1), refers to insecurity in physical, social, and economic access to sufficient, safe, and nutritious food supporting an active and healthy life. It can be a cause of hunger, undernourishment, and malnutrition (6). Dietary diversity, “a qualitative measure of food consumption that reflects household access to a variety of foods, and is also a proxy for nutrient adequacy of the diet of individuals” (7), is often used as a proxy measure of dietary quality with respect to intake of micronutrients and an indicator of food security (8). This article draws on insights from an intervention with indigenous communities in Bihar, India, one of the endemically malnourished populations and regions of South Asia (9), to examine its potential impact on dietary diversity.

Santhals are one of the largest tribal groups in Eastern India, with their native language Santhali recognized as an important language through its additio to the Consitution of India's Eighth Schedule in 2003. Select Santhal youth were trained and supported to make films about diverse local foods that were part of their cuisine and heritage. These films were published on their YouTube channel and screened by them in villages in the same *panchayats* (local government areas) to which they belonged. By enhancing knowledge about locally collected food and their nutritional values, the project activities sought to give visibility to indigenous knowledge and increase the participation of community members in moving toward food security. The activities also sought to challenge unequal power relations observed among women and men in Santhal communities (10). YouTube as a host medium was selected by the Youth Club as it was a familiar medium and provided easy access for viewers and filmmakers at any skill level.

Research suggests that filmmaking has emerged as a democratizing tool, an effective method for giving voice to the marginalized (11–14) especially in creating well-received health communication and community engagement (11, 15–18). Participatory filmmaking relates to a collaborative process in which issues faced by participants are investigated by way of producing a film that informs and moves the community toward collective action and exposes covert social relations (19). Baumann et al. (20) pointed to the strengths of participatory filmmaking: the embodiment of experience, such as the use of participants’ voice and language; the capturing of spatial, temporal, and sensory data; and community engagement, yielding various audience interpretations of the film. Several studies have illustrated such embodiment, including Baumhardt et al.'s (15) on the practices of growing food in a field, its storage, and consumption in the context of climate change in Malawi.

Apart from forming valuable communication tools for spreading information in rural populations with limited exposure to formal education, these films helped gain community approval (21, 22). Critical thinking is another desired effect of participatory filmmaking that has been demonstrated in the context of health and nutrition (11, 17, 18, 23, 24). For instance, in a study of Aboriginal youth who were supported to create a film about type 2 diabetes prevention (24), the experience of making and viewing the film was noted to stimulate critical thinking and ongoing conversations on the social and political factors affecting the community members’ nutrition and health behavior.

The notions and practices of community participation more broadly are well documented, with roots in the 1960s and 1970s,  in community development, emancipatory participation, and liberation theology (25, 26). With the rapid adoption of the language of participatory development by a range of stakeholders, including multilateral and bilateral agencies and nation-states, skepticism about its empowering and transformative potential grew by the 1990s (25, 27, 28). A major critique related to the failure of participatory development to adequately engage with issues of power and politics, making it yet another technical approach to development (25, 29). In this article, we place power relations at the center of our analysis of participation, focusing on the “exercise of agency in relation to development” (25), with marginalized groups as active claims-making agents.

A second critique of participatory approaches points to the need for evidence to demonstrate outcomes, rather than considering these as a given, following the adoption of bottom-up, people-centric, and process-oriented approaches (in contrast to government-led blueprint approaches). Hickey and Mohan (25) suggested that it is important to not only distinguish between diverse kinds of participatory approaches but also expand the locus of transformation beyond the individual and local to encompass the institutional and structural components of a society. Project design thus needs to provide spaces or socially constructed sites *1*) that can challenge the production/reproduction of unequal power relations, *2*) that are cognizant of whose perspectives are being communicated, and *3*) that provide a voice to marginalized populations (25, 29–31). This was reflected in a review article on the use of digital storytelling, typically a 2- to 5-min audiovisual clip combining photographs with voiceover narration (and other audio if desired) (2), as a liberal arts method in research with mostly marginalized groups (32). The advantages of this method have been shown to outweigh the disadvantages as explored in the context of mainly developed countries (32). How this method can be utilized in the context of developing countries is yet in its infancy.

Our article aims to contribute to this debate on the transformative potential of participatory approaches to improve food systems, dietary diversity, and nutrition, drawing on insights from a project intervention CHIRAG (Creative Hub for Innovation & Reciprocal Research and Action for Gender Equality, meaning lamp in Hindi) that sought to engage Santhal youth in a process of documenting and sharing knowledge about a range of locally available foods in their communities, including forest foods. Whereas the contribution of common-pool resources to biodiversity is recognized, their contribution to nutritional outcomes remain underresearched (20, 33), with most studies focusing on cultivated crops and their nutritional outcomes. In the context of South Asia, Kadiyala et al. (34) have outlined multiple pathways and indeed disconnects between agriculture and nutrition, as these are mediated by climate change and uncertain production, market price variability, and the consequent uncertainty of income. The authors also highlight gender relations in terms of women's control over decision making, their time trade-offs between agricultural work and care, and their health, as important determinants of nutritional outcomes (see also 35, 36). Although women are generally responsible for the collection and processing of all foods, in the case of forest foods or locally collected foods, so are children and men across many indigenous communities in India and indeed globally (37). Apart from an emphasis on building knowledge and awareness about local foods, it is imperative that nutrition literacy target children and men, not just women, as it has been shown that food security is improved when nutrition information is provided to both male and female heads of households (38). Thus, collective access to nutrition literacy is more effective than only women's access to it. Through our research, we have sought to better understand the potential of participatory filmmaking in broad-based sharing of traditional food knowledge and practices within communities to improve dietary diversity and ultimately nutritional outcomes by way of enhanced nutrition literacy.

## Methods

To ensure the voice and representation of the community members, a participatory action research approach was designed, which helped identify relations of power and authority; built knowledge-sharing platforms; and addressed inequities due to temporal, spatial, and social structures, particularly those of gender and ethnicity (29). Special attention was given to ensure that outsider biases, such as geographic accessibility (spatial bias), person, season, or project bias (39), did not creep into the selection of the participant youth volunteers. Although they were provided with some orientation around sustainable food systems, along with the provision of a tool for producing content for a public medium—namely, filmmaking for a YouTube channel (cf. 40)—they were free to choose what, where, how, and when to film, drawing on the critical principle of creating space for their voice (29, 40). The participants, specifically the local youth, made films about their own lives and milieu, with a view to highlight their food preferences and cultures. These films were screened in small gatherings of fellow villagers to generate a critical discussion. They were also shared more widely on a public YouTube channel.

Institutional Review Board clearances were secured for the researchers and filmmakers from the University of East Anglia's Development Ethics Committee, Norwich, United Kingdom, and informed consent was sought from the participants in the films and the viewers. Each participant read the consent form (or it was read to him or her), and agreed explicitly to participation in the film or discussions, interviews, and collection of observations. Consent release forms were obtained from all participants featured in the films. The purpose of the Youth Club being to publish and spread information about their cultural and food practices, participants consented to their films being uploaded for public viewing on the YouTube channel that they set up for this purpose. The informed consent for the student filmmakers also included agreement on the further use of the material and films. For instance, one of their films (No. 47) was one of two selected globally for screening and discussion at the Table-to-Farm Video Challenge (Youth Alliance for Zero Hunger; UN Food Systems Summit 2021). Another film was selected for the Science Film Festival 2021 and screened across South Asia. Some of the films have been used for educational purposes, including as resource material for an online course on “Creative Communication, Extension Education and Sustainable Development” in collaboration with the Indira Gandhi National Open University. During the project period, the filmmakers were provided a small monetary honorarium; however, the greater reward for them was affirmation and recognition of their knowledges and practices. These filmmakers belonged to a marginalized indigenous community, and this wider use and recognition were central to their identity, their sense of self, and their empowerment; hence, they chose to present their material on a public platform, with data not anonymized.

Our intervention was introduced in the Jamui district of Bihar, among the worst-off districts in India in terms of meeting the SDGs, including SDG2. In the selected block, 17% of the 1.76 million people are indigenous, mostly Santhals, as opposed to 4.5% for the district (41). Although they reside near forests, with access to forest foods documented as having high nutritive value when compared with other foods (42), these communities are nevertheless more vulnerable to food and nutrition insecurity compared with their rural counterparts (43, 44). In terms of nutritional status, 44% of indigenous children aged <5 y are stunted, 45% are underweight, and 27% wasted (45). Less than 6% of young children, women, and migrating men have a diet that fulfills their minimum nutritional requirements (44), a key constraint being the high cost of nutritious food and low affordability of healthy diets (46).

The Santhal youth participants in this study are members of a Santhal youth collective organized by a leading Indian nongovernmental organization. Sixteen interested youth filmmakers from three of the organization's field locations in the Santhal Parganas division (an administrative division constituted of 6 districts (Dumka, Deogarh, Jamtara, Pakur, Godda and Sahibganj) in the neighboring state of Jharkhand) were identified in November 2019 and introduced to the idea of participatory filmmaking. All 16 participants were trained in a filmography workshop in March 2020, conducted by a UK-based film studies professor. They learned how to use digital video cameras, construct a story, and film and edit. Of the initial 16 participants, 10 continued with the process, all as members of a club interested in promoting youth engagement in local development and change. This final group included 3 women and 7 men from the selected district; all were 18–25 y old, except one who was 45 y. The 45-y-old was an integral part of the club, a single man who galvanized the youth. The CHIRAG project built on this existing engagement with youth to discuss issues of food security and local dietary diversity, encouraging them to engage with this theme as an important element of indigenous knowledge, culture, and indeed improved health and well-being.

Initially, filmmaking was slow due to the nationwide lockdown imposed by the government on 25 March 2020, owing to the COVID-19 pandemic, the infectious disease caused by the SARS-CoV-2 virus (47). Once restrictions were eased, the nongovernmental organization's research team conducted refresher training (July and September 2020), followed by a second filmography workshop by an Indian filmmaker in December 2020 and February 2021, raising issues of language, gaze, and the politics of representation. Fortnightly meetings were held with the youth filmmakers to share lessons and provide feedback. Additional training was conducted to respond to specific needs, such as video editing, scene writing, and shooting (March 2021 and June 2021). The project provided the filmmakers with filming and editing equipment and access to the Internet for editing purposes in centralized locations, as connections were erratic in the region. Given the extensive use of mobile technology, especially by young people, to access social media, the youth chose YouTube as their preferred channel for dissemination.

The Youth Club's YouTube channel hosted 70 videos by August 2021. Our thematic content analysis (48) focused on 49 videos that were fully or jointly produced by the youth filmmakers (full list in **Supplemental Table 1**). We assessed the shifts from before February 2020 (baseline; phase 1) to the period from February to December 2020 (posttraining 2020; phase 2) and then after January 2021 (posttraining 2021; phase 3). To analyze the films, a table including the list of films was created. Variables such as presentation, filming techniques, main topics covered, issues raised, length of each film, number of views, settings, actors, and sound effects were included with additional comments, as needed, for each film. A constant comparative method (49) was used to identify common themes across the films. To ensure the specificity and inclusivity of each identified theme, subissues were merged and a distinct number allocated to each major theme. This created the thematic template (50) for extracting supportive data from interviews and focus groups.

Additional data were collected from in-depth semistructured interviews with the 10 filmmakers to explore their experiences and their choice of topics and formats. In addition, data were collected from focus group discussions in Santhali with >100 viewers who attended 4 of the 28 film screenings. The objective was to explore the effect of the viewings on the audience. Data were also drawn from a detailed process documentation system set up for the project, which included the collection of observations of the trainers and the filmmakers.

## Results

**FIGURE 1:**
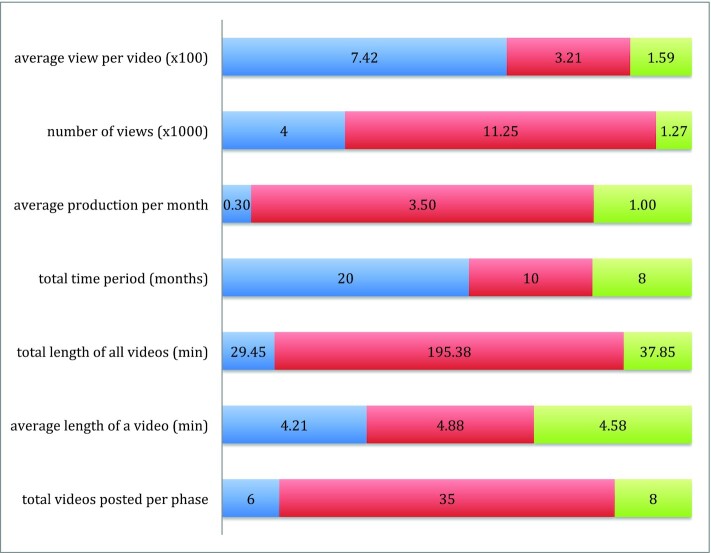
Quantitative data on films produced by the filmmakers. 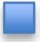
 Phase 1: pretraining. 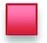
 Phase 2: between trainings 1 and 2. 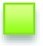
 Phase 3: post–training 2.

The YouTube channel had 546 subscribers as of May 23, 2022. **Figure 1** provides an overview of the type and number of films produced and the related public viewership on a temporal axis. A majority of the films focused on sustainable food systems, with topics including knowledge about edible plants, insects, and rodents; skills in foraging and preparing food; awareness of the benefits of the food; and sustainability issues across the traditional food system of the Santhal communities. Local foods included specific tree leaves, mushrooms, a type of rodent, snails, small water-dwelling animals lower on the food chain (e.g., small fish and crabs), fruits, and many types of leafy greens. Sustainable pest management was also highlighted, such as making traps to capture rats in agricultural fields. Interestingly, the filmmakers expanded the concept of sustainability beyond food systems to show, for instance, how a grassy weed was woven into brooms for use in households and to earn some extra cash to support household food security.

Knowledge transfer across generations was shown multiple times in the films. When asked how an interviewee learned a skill or learned about eating a particular uncultivated food, the most common responses were “my grandparents and parents” (video 35), “my grandfather” (videos 26 and 39), and “My father taught me to make this” (video 33). Film 29 captures the process of this knowledge transition from an elderly guardian to young boys when collecting wild yam from a forest. The guardian shares how his grandfather taught him to identify and collect a variety of yams, thus establishing the practice as a traditional one to the audience. It must be mentioned that the language used in these films is Santhali with English subtitles.

**FIGURE 2:**
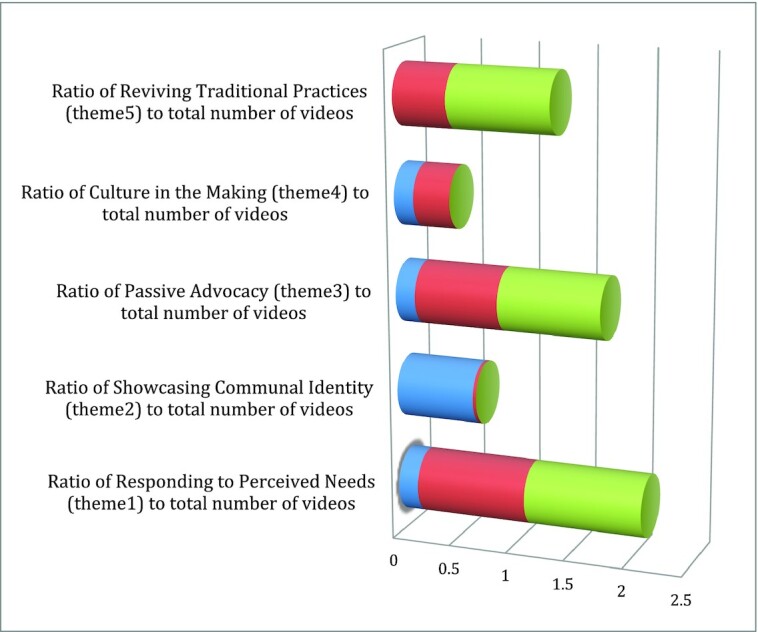
Ratio of identified themes to the total number of videos posted in each training. 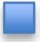
 Phase 1: pretraining. 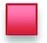
 Phase 2: between trainings 1 and 2. 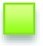
 Phase 3: post–training 2.

Several themes emerged from the analysis of the films (**Figure 2**), and these are discussed in turn, alongside related reflections of the filmmakers on their journey. We find a shift from films showcasing community identity in phase 1 to responding to emergent needs, as well as critically reflecting on traditional practices and external threats, in phases 2 and 3, as the filmmakers gained confidence in their skills, ideas, and authorship.

### Theme 1: Responding to perceived needs

A large number of films (42 of 49) were produced in response to an everyday community need, be it the collection of nutritious uncultivated foods or the making of tools with local materials, such as rat traps and brooms. Four videos demonstrated the use of technology—for instance, how to use mobile interactive voice response technology to obtain information on COVID-19 prevention measures. The occurrence of this theme is higher in phase 2 and phase 3 compared with the prepandemic phase 1, reflecting the breakdown in supply chains and access to external resources but equally the additional training and discussion with the filmmakers on food system-related issues.

An 18-y-old filmmaker said that she was initially embarrassed, as women do not hold cameras or make movies and it is not acceptable for them to appear in the film. “After shooting the film I sighed deeply as I had found my vocation. I chose acacia (video 8) for my film because there was the lockdown and we could not buy soap in the market. We use acacia as a hand wash traditionally. I then decided to make this film so that others got to know about it. How else would we manage during the lockdown? Anyway, washing hands is very important for us to emphasize.” The filmmaking here resulted in strengthening the voice of the young filmmakers, who would otherwise be unheard and unable to respond to the needs of their communities. It was also a means of providing timely information, showcasing the skills and knowledge of the local people, and presenting inspiring stories to motivate the adoption of sustainable and beneficial practices by the larger community.

### Theme 2: Showcasing communal identity

In phase 1, the filmmakers covered topics related mainly to their culture and identity and the activities embedded deeply in their lives. For example, the harvest festival Sohrai was a major theme (video 4), as were commonly consumed foods with ingredients both foraged and purchased from the market. *Seem peetha*—a type of bread made of spiced cooked chicken and flour sandwiched in the *sal* leaf and baked in fire—was the subject of five films. In the words of a 21-y-old male filmmaker, “I have understood that I can make a small film and take the story of the village home to the world through the film. There are a lot of activities in our society that I want to bring to the world.” An 18-y-old female added, “My grandmother becomes very happy whenever I ask her about local food and culture. She says that during her childhood there were no phones or electronic media, or social media where she could have shared her knowledge. So it's a good thing that her granddaughter is trying to preserve what is their own by making films around the food they get from the forest and reach out to many people around the world.” The filmmaking journey, particularly in phase 1, served the purpose of showcasing Santhal identity to the external world.

### Theme 3: Passive advocacy

The act of holding a camera, filming, and telling their stories was a key theme that emerged from the interviews conducted with the filmmakers: “There is a perception among the villagers that we as in villagers cannot produce films or shoot photographs, but this perception was broken when I stood in front of them with a camera in my hand and was making films about local foods” (male, 45 y). Here, the filmmakers are consciously attempting to voice a critical issue, whether endemic malnutrition or vast deforestation, as a call to policy makers for remedial action.

However, as no direct statements were made addressing government officials and the films were not used for any campaigns or protests, they appear to form a passive advocacy tool. They invite empathy and understanding and ask for support to the Youth Club to strengthen the voice of the rural communities. Such films are much higher in number in phases 2 and 3, indicating the strengthening voice of the filmmakers as they progressed from simply displaying how things were and showcasing communal identity, the highest occurring theme in phase 1, to raising flags to an external audience about overarching issues that reduce opportunities for self-sustenance.

### Theme 4: Culture in the making

Culture in the making refers to changes or modifications to certain aspects of culture. A clear example is the film about young boys hunting and cooking bamboo tree-dwelling rats in a forest (*banwar peetha*, video 47). Although narrations by a young boy in the Santhali language give a sense of representation of all Santhal children, the practice of hunting and consuming these rats is practiced only by boys outside their homes in the forest and open fields. The nutritional profile of this food and its contribution to good health are highlighted in the film. Toward the end (5:12–5:23 min:s), the narrator in the film poses a critical question to his community: “Let us think if there are so many benefits in eating *banwar* [a type of rat], can women and girls not cook it in their home and eat it too?” The filmmakers have identified a gender discriminatory norm and, through this film, are attempting to reconstruct this cultural norm so that girls are not deprived of the benefits of this source of nutrition.

The division of food-related labor along gender lines is apparent in several films, although not always to the disadvantage of women. For example, whereas catching fish and crabs from fields during the monsoon season or climbing tall trees to forage for fruits is done by boys (videos 10 and 23), when the collected food is brought back home, it is prepared and served to all members of the household by the woman of the house. These films demonstrate the growing power of participatory filmmaking in reflecting on cultural practices and initiating discussions on positive modifications that can support gender equality in food, nutrition, and health.

### Theme 5: Reviving traditional practices

Reviving traditional practices was defined as the building of pride of the Santhal community through a revival or strengthening of a range of traditional activities and reflected in 23 of the 49 films, most of them produced in phases 2 and 3. For example, in the film about wild yam collection (video 29), a young boy says that he has never eaten wild yams, whereas at the end of the film, another boy says that he would like to continue foraging for wild yams like his father. The importance of reviving traditional food practices is displayed in a phase 2 film on banyan tree fruit collection (video 26), where a boy interviewed said, “In the old times when people didn't have rice, they used to eat fruits like *bade bili* for survival.” This showed how survival food had changed to rice, which, however, has much lower nutritional value than the *bade bili* fruit. Subsequent films continue to display this theme in their content, linked to the reflection on the need to return to traditional food choices for improving nutritional outcomes.

The filmmakers’ shifting orientation is expressed by one of the filmmakers (female, 22 y), who spoke about her satisfaction at enhancing community interest in local food: “I feel good. Information is being spread about our shared culture and traditions, what we eat and drink, and how we live. All our people were remembering old things and telling each other about themselves.” The 45-y-old male participant further noted, “There is a difference between knowing and adopting. Even if I know traditional practices, I may not practice them myself and may not share the knowledge with anyone or encourage others to practice. Then what is the use of knowing such rich facts? In this world of modernization where chemical medicines are readily accepted, our knowledge is getting lost and our culture along with it.”

## Discussion

This article contributes to the debate around the use of participatory approaches—in this case, giving a tool in the form of cameras to the most marginalized to revitalize traditional foods (mostly nonmarket and even noncultivated) as a response to food insecurity and possibly malnutrition. Specifically, this article discusses a participatory action research project, involving participatory film-making by indigenous youth to document and transmit traditional knowledge about locally available foods, and its impact in improving dietary diversity. In this process, power relations, especially gender based, are examined and challenged across the food system. The shift in themes across phases 1–3 is not surprising as the filmmakers were initially developing technical skills and focusing more on showcasing their common cultural practices. By phase 3, they began to reflect on aspects of their culture that needed to be preserved, revived, or modified. Overall, the quality of the films produced improved over the three phases, perhaps a result of continual technical training and their growing experience as filmmakers, making the delivery of messages more impactful.

The community audiences had positive responses to seeing films about their own contexts and lives and largely appreciated not just the information received but its relatability and relevance. The following quote is typical: “These films have given us different information. We are happy that they are working on Santhali culture” (group discussion, villages B and L). The cooking scenes showcasing the skills and knowledge of the local people especially resonated with the women. They were delighted that “our food preparation recipe is being shown in a film. When we search on YouTube to cook something, there is always a modern kitchen, how villagers cook is missing in social media and TV serials. We now have a platform for each other and others like us to share new and healthy recipes” (group discussion, village A).

A key element of participatory action research is providing the space for action and reflection (51). A team member observed, “The process helped people reflect. Whatever was shown on the screen, they began speaking to each other about it, recalling what they had seen.” Although they noted that forest foods were liked by children, adults, and older people, they usually did not speak about them due to a sense of shame associated with such foods. As one member noted, “it is hardly talked about by us. Why? Because when people outside our community talk about it, they say that Adivasis are primitive, they eat wild foods. We do eat these, but we do not speak about it” (group discussion, villages B and L). This demonstrates the liberating aspect of the project where the participants had the opportunity to talk freely in their own language about their own perspectives and practices without fearing alienation by the dominant culture. In a study on the Aboriginals in Australia too, the experience of filmmaking was seen as liberating the participants from the constraints of language and health education communication used by health care professionals (18).

At the same time, audience reflection on its culture helped to identify practices that needed to be preserved, revived, or indeed modified. There were many suggestions for films that could document other food items and recipes, especially those linked to health and nutrition. For example, “Like *Aata ser* we also get *kappu* in the jungle [wild fruit]. We soak it in water for 12 hours and drink the water to reduce body ache and to relieve flatulence. We need a film on *kappu* also, so that people know its benefits and are willing to do the hard work to collect it. It is very beneficial for us Santhals” (group discussion, village R). In the group discussion in village A, the women specifically mentioned *jamdo ara* and *rano bayya* (leafy greens) as useful in treating vaginal infections and *chakanda ara* (*senna* in English) to stimulate bowel movements and alleviate constipation. Women also wanted films to focus on children, who went to the forest to play and picked up food items along the way, eating them without washing. Mothers suggested training them in washing, cleaning, safety, and nutrition through the medium of films.

Rao (51), in an ethnography of the Santhals, found that whereas women were not afraid to speak to government functionaries, it was the latter who chose not to speak to them but only to their men. The key issue seemed to be one of language. Here, as the films were in Santhali and the filmmakers were from their own community, the women appear to have no difficulties in speaking their minds. When assuming women to be passive, one often misses critical power relations embedded in language, which impose silence on women. In the Santhal Parganas, until a decade ago, >60% of Santhal women were monolingual in Santhali (51). Yet, women did note that they felt somewhat constrained to go into details, especially about health issues, with the male filmmakers. There is clearly a need for further work by the women filmmakers to give adequate recognition to women's knowledge, especially around the medicinal properties of different plants. When handled with sensitivity, filmmaking can thus have democratizing effects, in this case on marginalized nonliterate women, as seen also in other contexts (24, 52).

The dialogue between the filmmakers and the audiences points to the creation of a space where people's voice and agency have the potential to be strengthened. A trusting relationship has been built between the young filmmakers and their community audiences, with the latter actively engaging in the process of viewing the films and reflecting on them, suggesting improvements in the films, different ways of presenting the ideas, or even new themes for future films. One finds, even within a short period, a sense of ownership of the agenda, making their voice count in a portrayal of their society and its food cultures. A pre– and post–film viewing survey in the four villages showed an overall increase of 55% in the audience's willingness to adopt new practices. Participatory filmmaking has been noted to meet high levels of approval from participants elsewhere too (18, 21).

Finally, this process has led to a shift in terms of identity, greater confidence in one's sense of self, a move from shame to confidence, reflecting a process of empowerment (53) among the filmmakers, the community, but equally our project team members. One of them reflected, “As a tribal person, this was my first experience of building on my background and speaking about it. It was a big challenge for me too, living in an urban area. I too had developed a mindset that we should not eat wild food, as it is considered backward. But as I went through the process, I felt interested, as were the filmmakers. We connected with our culture and talked about it.”

In conclusion, filmmaking has the potential to not just document but create space for dialogue within and between epistemic communities: the external “scientific” community and the indigenous “knowledge” community. As films are made, they challenge some of the biases and notions of “shame,” “backwardness,” or indeed “modernity.” In creating a more respectful dialogue between different actors, we started with the voice of the most marginalized. Follow-up action with the participants and the residents in the three *panchayats* (local government areas) was facilitated by the project team. There were questions raised and experiences and knowledge shared as related to diets, malnutrition, and food preferences and availabilities. The communities were linked to an interactive voice response-based mobile call-in system, also run by the project team. A nutritional assessment of the Santhal recipes discussed in the films is underway to better understand their macro- and micronutrient contributions to diets. Finally and perhaps most important, local media coverage about the Santhal youth filmmakers has brought them to the attention of senior political leaders, acknowledging the potential of this medium for reviving but also modifying indigenous practices through a dialogical process.

This points back to the debates on addressing the challenges of equity and inclusion within participatory approaches. How can we ensure a participatory process that gives space and voice to all stakeholders, not just hearing the voices and versions of the vocal few (5,6, 54)? The role of self-expression, especially for indigenous communities, is important, given the discourse of historical marginalization and adverse incorporation (55). Without participation in governance and the political space, transformative change alongside improved health and nutritional outcomes will be hard to achieve. The use of the YouTube channel as a digital “space” created by the participants to display their films conferred them an opportunity to set the agenda in the restructured power relations where they can speak without tangible (physical) or intangible (psychological) interruptions. Although certain Santhal dietary practices are mocked in mainstream spaces, constructed as “wild” and “backward,” filmmaking has given them the power to resist such social constructions that have led to the invisibility of their food practices and cultures. Even though it is too soon to comment on the actual nutritional outcomes of this intervention, making the local diversity in food and consumption practices visible itself provides room for change.

## Supplementary Material

nzac114_Supplemental_File

## Data Availability

The films are freely available on the Lahanti Club YouTube channel.

## Notes

Data collection was supported by an EPSRC Global Research Translation Award (EP/T015411/1UKRI; “Meeting the SDGs”). The funder had no involvement in the study design, collection, and analysis of data and posed no restrictions regarding publication.

Author disclosures: The authors report no conflicts of interest.

We have secured ethics approval from the University of East Anglia Development Ethics Committee and followed the protocols of consent, anonymity, and confidentiality of all respondents, with the exception of individuals who posted their videos on public platforms such as “YouTube” and public screening events.

Supplemental Table 1 is available from the “Supplementary data” link in the online posting of the article and from the same link in the online table of contents at https://academic.oup.com/cdn.
