# Supplementary material for: Cameras in the Hands of Indigenous Youth: Participation, Films, and Nutrition in India
Source: Curr Dev Nutr. 2022 Aug 19;6(8):nzac114. doi: 10.1093/cdn/nzac114 (PMC9388318; doi:10.1093/cdn/nzac114)
Supplement: nzac114_Supplemental_File [file nzac114_supplemental_file.docx]

Supplementary Material

**Table 1.** Videos produced and screened by Lahanti Club youth

| **Period** | **No.** | **Film Title** | **No. of Online Views** | **Link to Videos on Youtube** |
| --- | --- | --- | --- | --- |
| Prior to any training | 1 | Santhal Dish- Seem Peetha | 2,978 | [https://www.Youtube .com/watch?v=gK41YztHh78&t=389s](https://www.youtube.com/watch?v=gK41YztHh78&t=389s) |
| (Phase I) | 2 | Santhal Life | 148 | [https://www.Youtube .com/watch?v=UjHIz-zyhYg](https://www.youtube.com/watch?v=UjHIz-zyhYg) |
|  | 3 | Santhal Culture and Creative Media | 299 | [https://www.Youtube .com/watch?v=0X5SCE2rpuE&t=77s](https://www.youtube.com/watch?v=0X5SCE2rpuE&t=77s) |
|  | 4 | Sohrai | 197 | [https://www.Youtube .com/watch?v=0fo4GWRZFOI](https://www.youtube.com/watch?v=0fo4GWRZFOI) |
|  | 5 | Making Nukkad Natak | 502 | [https://www.Youtube .com/watch?v=z4lvb5sZ6IA](https://www.youtube.com/watch?v=z4lvb5sZ6IA) |
|  | 6 | Roasted Godo | 210 | [https://www.Youtube .com/watch?v=TTFpBMGJIEI](https://www.youtube.com/watch?v=TTFpBMGJIEI) |
|  | 7 | Episode 1: Festival, Food and Nutrition | 437 | [https://www.Youtube .com/watch?v=xZ_KE0c4t_s](https://www.youtube.com/watch?v=xZ_KE0c4t_s) |
| Between trainings #1 & # 2 | 8 | Sustainable Practices: Part-One Acacia | 315 | [https://www.Youtube .com/watch?v=PwD68G4EeQE](https://www.youtube.com/watch?v=PwD68G4EeQE) |
| (Phase II) | 9 | Mud wall painting for sustainable food system | 216 | [https://www.Youtube .com/watch?v=4KJN067fFy0](https://www.youtube.com/watch?v=4KJN067fFy0) |
|  | 10 | 'Heshak Ara'- Foraging Food item | 585 | [https://www.Youtube .com/watch?v=Ueza_qXvwxQ](https://www.youtube.com/watch?v=Ueza_qXvwxQ) |
|  | 11 | Chiraagvaani Tutorial | 58 | [https://www.Youtube .com/watch?v=PsSy2Iv_ukw](https://www.youtube.com/watch?v=PsSy2Iv_ukw) |
|  | 12 | Chiraagvaani Tutorial | 151 | [https://www.Youtube .com/watch?v=PI8Fn05fHn4](https://www.youtube.com/watch?v=PI8Fn05fHn4) |
|  | 13 | Chiraag, Tutorial 2 | 116 | [https://www.Youtube .com/watch?v=Zj4i4BGAqLQ](https://www.youtube.com/watch?v=Zj4i4BGAqLQ) |
|  | 14 | Chiraagvaani Santhali Tutorial | 82 | [https://www.Youtube .com/watch?v=3sksEyjR3Fw](https://www.youtube.com/watch?v=3sksEyjR3Fw) |
|  | 15 | Santhali Tutorial 2 | 243 | [https://www.Youtube .com/watch?v=HgUSr5qS6ZA](https://www.youtube.com/watch?v=HgUSr5qS6ZA) |
|  | 16 | Learning by Listening | 437 | [https://www.Youtube .com/watch?v=hWv9s0AQ0fk](https://www.youtube.com/watch?v=hWv9s0AQ0fk) |
|  | 17 | Forage Food- Lowa Billi- Foraging Skills of Santhal Community | 398 | [https://www.Youtube .com/watch?v=UuuOO08mqUk](https://www.youtube.com/watch?v=UuuOO08mqUk) |
|  | 18 | Call for Volunteers II Education initiative in COVID-19 situation | 211 | [https://www.Youtube .com/watch?v=ra3Uz1zBnTY](https://www.youtube.com/watch?v=ra3Uz1zBnTY) |
|  | 19 | Sacred Fruit- Soso | 490 | [https://www.Youtube .com/watch?v=_vFuhhnwDGc&t=93s](https://www.youtube.com/watch?v=_vFuhhnwDGc&t=93s) |
|  | 20 | Haat Baha- Food, medicine and more! | 497 | [https://www.Youtube .com/watch?v=tyv0cHWnang](https://www.youtube.com/watch?v=tyv0cHWnang) |
|  | 21 | Education innovation. Lahanti Club | 401 | [https://www.Youtube .com/watch?v=xGqoyF6im5k](https://www.youtube.com/watch?v=xGqoyF6im5k) |
|  | 22 | Sing Ara!- A green treasure for your health | 271 | [https://www.Youtube .com/watch?v=Ulyie3AVQI0](https://www.youtube.com/watch?v=Ulyie3AVQI0) |
|  | 23 | Tiril Bili- Local fruits | 803 | [https://www.Youtube .com/watch?v=vnBbuasnfsU](https://www.youtube.com/watch?v=vnBbuasnfsU) |
|  | 24 | Rokoy- Local delicacy for sustainable food system! | 306 | [https://www.Youtube .com/watch?v=W3azVyNh2FY&t=69s](https://www.youtube.com/watch?v=W3azVyNh2FY&t=69s) |
|  | 25 | Mung Ara- Sustainable Food System | 383 | [https://www.Youtube .com/watch?v=3GED5F3muAY](https://www.youtube.com/watch?v=3GED5F3muAY) |
|  | 26 | Bade Billi- Fruit of Banyan Tree | 1022 | [https://www.Youtube .com/watch?v=7pdyohwE3NY&t=107s](https://www.youtube.com/watch?v=7pdyohwE3NY&t=107s) |
|  | 27 | Ek Palle Ka Taraju- Science in indigenous practice | 707 | [https://www.Youtube .com/watch?v=7J6xVYGf3O8](https://www.youtube.com/watch?v=7J6xVYGf3O8) |
|  | 28 | Goddo Phasi- Indigenous Rat Trap | 291 | [https://www.Youtube .com/watch?v=JVvOABH5mTg](https://www.youtube.com/watch?v=JVvOABH5mTg) |
|  | 29 | Aat Aser \|\| Food from Forest | 302 | [https://www.Youtube .com/watch?v=nPe-IJmiYVk&t=75s](https://www.youtube.com/watch?v=nPe-IJmiYVk&t=75s) |
|  | 30 | Moringa Oleifera leaves \| Munga Pakori \| Sustainable Food System | 152 | [https://www.Youtube .com/watch?v=qWu3goXjFZY](https://www.youtube.com/watch?v=qWu3goXjFZY) |
|  | 40 | Ortua Oo \|\| Food During Covid-19 II Food From Forest | 369 | [https://www.Youtube .com/watch?v=kSkTtlCc-NE](https://www.youtube.com/watch?v=kSkTtlCc-NE) |
|  | 41 | Saga: Jono (Broom) | 116 | [https://www.Youtube .com/watch?v=6oUL0HnmmSU](https://www.youtube.com/watch?v=6oUL0HnmmSU) |
| During & Post-training #3 | 42 | Katkom Chutney (Crab) (Brachyura) \| Sustainable Food System | 120 | [https://www.Youtube .com/watch?v=F11I-CMs80o&t=277s](https://www.youtube.com/watch?v=F11I-CMs80o&t=277s) |
| (Phase III) | 43 | Hau Chatni (Cheeti Ki Chatni) \| Sustainable food system | 133 | [https://www.Youtube .com/watch?v=qBs-icB0qJo&t=1s](https://www.youtube.com/watch?v=qBs-icB0qJo&t=1s) |
|  | 44 | Dhurup Ara\|Sustainable Food System | 112 | [https://www.Youtube .com/watch?v=EfcaiAjCBF8&t=3s](https://www.youtube.com/watch?v=EfcaiAjCBF8&t=3s) |
|  | 45 | TITUA ARA\|Sustainable Food System | 65 | [https://www.Youtube .com/watch?v=pmKzGBD161Y](https://www.youtube.com/watch?v=pmKzGBD161Y) |
|  | 46 | Ohoy Ara \| Sustainable Food System | 121 | [https://www.Youtube .com/watch?v=WT1iiL31kJU](https://www.youtube.com/watch?v=WT1iiL31kJU) |
|  | 47 | Banwar Peetha\|Santhali recipe | 418 | [https://www.Youtube .com/watch?v=Kt8cHbpyick&t=324s](https://www.youtube.com/watch?v=Kt8cHbpyick&t=324s) |
|  | 48 | Kadha\|Immunity Booster\|Helpful recipe during COVID-19 Pandemic | 156 | [https://www.Youtube .com/watch?v=iMzsvRPGa-o&t=31s](https://www.youtube.com/watch?v=iMzsvRPGa-o&t=31s) |
|  | 49 | Soso/Bhelwa\|Local Medicine for cold & cough | 148 | https://www.Youtube .com/watch?v=Ey-kiXIuIPk |

^[1]^ Santhal is a native ethnic group in India. Their native language is Santhali.

^[2]^ CHIRAG meaning lamp in Hindi, is an acronym for Creative Hub for Innovation & Reciprocal Research and Action for Gender Equality.
